# Case report: persistently seronegative neuroborreliosis in an immunocompromised patient

**DOI:** 10.1186/s12879-018-3273-8

**Published:** 2018-08-02

**Authors:** A. Wagemakers, M. C. Visser, B. de Wever, J. W. Hovius, N. W. C. J. van de Donk, E. J. Hendriks, L. Peferoen, F. F. Muller, C. W. Ang

**Affiliations:** 1 Department of medical microbiology, VU medical center, De Boelelaan 1117, Amsterdam, 1081 HV The Netherlands; 2 Department of neurology, VU medical center, De Boelelaan 1117, Amsterdam, 1081 HV The Netherlands; 3 Department of medical microbiology, Academic medical center, Meibergdreef 9, Amsterdam, 1105 AZ The Netherlands; 4 Department of internal medicine/Amsterdam multidisciplinary Lyme center, Academic medical center, Meibergdreef 9, Amsterdam, 1105 AZ The Netherlands; 5 Department of hematology, VU medical center, De Boelelaan 1117, Amsterdam, 1081 HV The Netherlands; 6 Department of radiology and nuclear medicine, VU medical center, De Boelelaan 1117, Amsterdam, 1081 HV The Netherlands; 7 Department of pathology, VU medical center, De Boelelaan 1117, Amsterdam, 1081 HV The Netherlands

**Keywords:** *Borrelia*, Neuroborreliosis, Serology, Diagnosis, Immunocompromised, Rituximab

## Abstract

**Background:**

Infection with *Borrelia burgdorferi* sensu lato complex (B. b. sl) spirochetes can cause Lyme borreliosis, manifesting as localized infection (e.g. erythema migrans) or disseminated disease (e.g. Lyme neuroborreliosis). Generally, patients with disseminated Lyme borreliosis will produce an antibody response several weeks post-infection. So far, no case of neuroborreliosis has been described with persistently negative serology one month after infection.

**Case presentation:**

We present a patient with a history of Mantle cell lymphoma and treatment with R-CHOP (rituximab, doxorubicine, vincristine, cyclofosfamide, prednisone), with a meningo-encephalitis, who was treated for a suspected lymphoma relapse. However, no malignant cells or other signs of malignancy were found, and microbial tests did not reveal any clues, including *Borrelia* serology. He did not recall being bitten by ticks, and a *Borrelia* PCR on CSF was negative. After spontaneous improvement of symptoms, he was discharged without definite diagnosis. Several weeks later, he was readmitted with a relapse of symptoms of meningo-encephalitis. This time however, a *Borrelia* PCR on CSF was positive, confirmed by two independent laboratories, and the patient received ceftriaxone upon which he partially recovered. Interestingly, during the diagnostic process of this exceptionally difficult case, a variety of different serological assays for *Borrelia* antibodies remained negative. Only P41 (flagellin) IgG was detected by blot and the Liaison IgG became equivocal 2 months after initial testing.

**Conclusions:**

To the best of our knowledge this is the first case of neuroborreliosis that is seronegative on repeated sera and multiple test modalities. This unique case demonstrates the difficulty to diagnose neuroborreliosis in severely immunocompromised patients. In this case, a delay in diagnosis was caused by broad differential diagnosis, an absent known history of tick bites, negative serology and the low sensitivity of PCR on CSF. Therefore, awareness of the diagnostic limitations to detect *Borrelia* infection in this specific patient category is warranted.

**Electronic supplementary material:**

The online version of this article (10.1186/s12879-018-3273-8) contains supplementary material, which is available to authorized users.

## Background

Neuroborreliosis is generally considered to be accompanied by positive serology for Lyme borreliosis. Only few studies have described true seronegative Lyme borreliosis after weeks of untreated disease, none of which excluded seropositivity in serologic tests outside those routinely used in their local diagnostic protocol. Here, we present an immunocompromised patient with neuroborreliosis who was seronegative in a wide array of serologic tests, even after several months of follow-up. Final diagnosis was established by *Borrelia* PCR on CSF after readmission, after being PCR negative upon initial presentation. This underscores both the potential of a true seronegative neuroborreliosis, as well as the difficulty to diagnose this rare phenomenon. However, the findings in this unique case can obviously not be extrapolated to regular patient categories.

## Case presentation

**Fig. 1:**
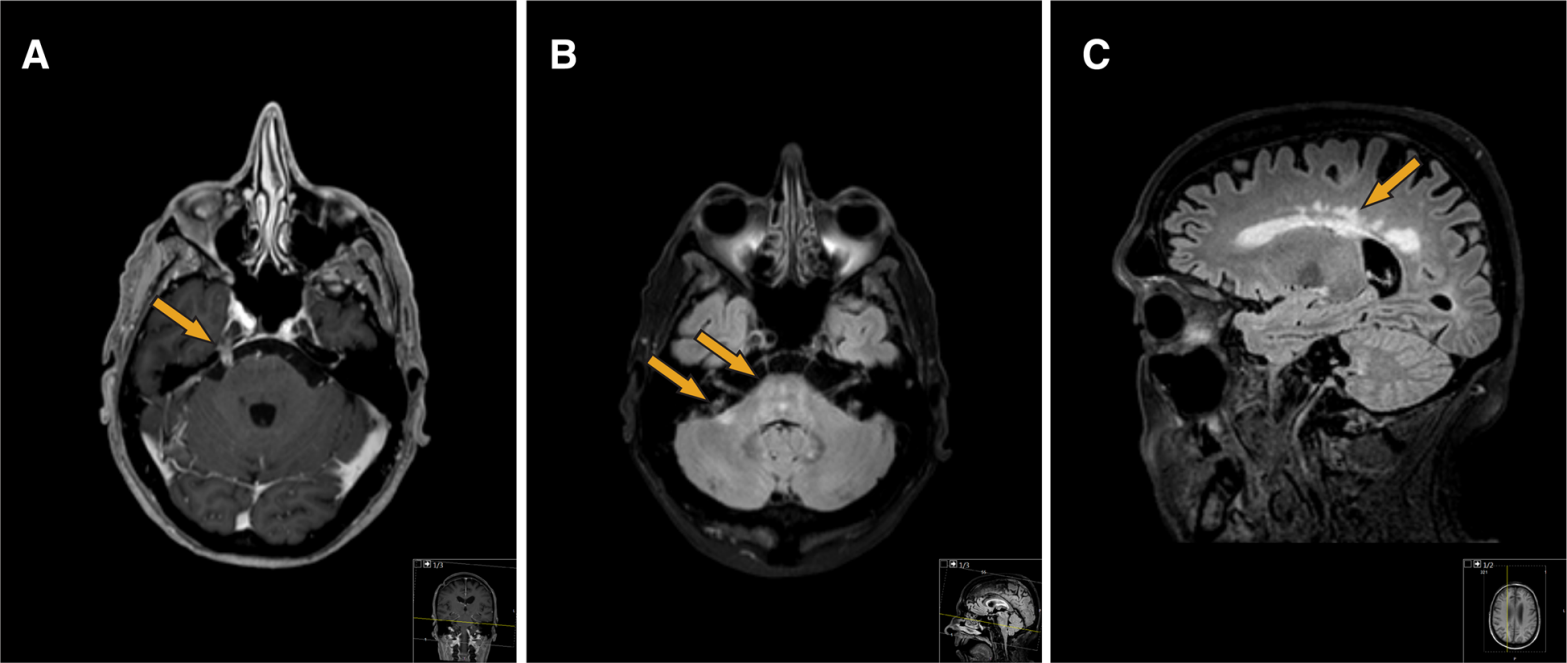
**a** 3D heavily T1-weighted gradient-echo pulse-sequence obtained after administration of intravenous gadolinium shows pathological contrast enhancement of the right trigeminal nerve, in the cisternal segment. **b** This 3D FLAIR turbo spin-echo pulse-sequence reveals hyper intense signal abnormalities in in the pons and right middle cerebellar peduncle. **c** Paramedian sagittal FLAIR sequence displaying periventricular confluent hyperintense signal abnormalities most certainly related to inflammation due to neuroborreliosis

A 70-year old man presented at his hematologist’s outpatient clinic on July 12th 2016 with 3 weeks of intermittent fever, myalgia, headaches, tinnitus and an exanthema, which had started under his left eye. His history revealed a mantle cell lymphoma (a B-cell non-Hodgkin’s lymphoma) in 2010, which was initially treated with R-CHOP (rituximab, cyclofosfamide, doxorubicine, vincristine and prednisolone), high dose cytarabine, and autologous stem cell transplantation. After achieving a complete response, he developed progressive disease in 2015, which was treated with R-CHOP. A second complete response was achieved, and he continued to receive rituximab maintenance every other month until July 2016. For his current symptoms, he was treated with azitromycin. A consulting dermatologist observed generalized nummular to palm-sized non-pruritic erythematous macules, sparing the foot soles, which was considered either a drug eruption or a para-infectious skin reaction. An abdominal skin biopsy revealed a non-specific chronic perivascular dermatitis, deemed consistent with a hypersensitivity response. A PCR on blood was negative for EBV and CMV, and a nasopharyngeal swab was negative for respiratory viruses. His next dose of rituximab was delayed until July 18th, at which point his erythema had resolved with only minor myalgias remaining. From July 22nd the patient started to experience a right-sided headache with right-sided rhinorrhea and a tearing eye, tinnitus of both ears and a different sensation for taste, as well as relapsing (sub)febrile episodes. From August 1st, he noted a left facial palsy, and on August 23rd the patient was admitted to the neurology ward with progressive symptoms. By this time, he had developed an unsteady broad-based gait, a mild action tremor, mild apathy, dysphagia and hearing loss. He had an erythema on his wrists and left elbow and had lost 6 kg. An MRI of his brain revealed periventricular white matter lesions, and pathological enhancement of multiple cranial nerves (bilateral trigeminal and vestibulocochlear nerves and right hypoglossal nerve, Fig. 1). These findings raised suspicion of a relapse of mantle cell lymphoma in the central nervous system, for which a diagnostic lumbar puncture was performed, and intrathecal prednisone combined with methotrexate was administered. A peripheral leukocyte differentiation was normal besides a lymphopenia (0,30 × 10^9^/ml), while total IgA (0.44 g/l), IgM (< 0.181 g/l) and IgG (2.92 g/l) were reduced (Table 1). The CSF showed 279 leukocytes/μl containing 60% non-clonal T-cells and only 0.001% non-clonal B-cells, and elevated protein (2.55 g/L) (Additional file 1: Table S1). Five days after the lumbar puncture and intrathecal chemotherapy the patient deteriorated with nausea, a nystagmus and progression of the unstable gait. An MRI was repeated and showed a small increase of basal and cerebellar leptomeningeal enhancement, as well as recent focal ischemia in the right medulla oblongata. Additional body CT and a PET-CT did not reveal lymphoma or pathological FDG absorption. Repeated cerebrospinal fluid (CSF) analysis including immunophenotyping did not reveal malignant cells, prompting a search for an alternative diagnosis.

**Table 1:** Overview of results from serology and molecular diagnostic tests for *Borrelia*

	26–08-2016 serum	31–08-2016 CSF	7–10-2016 CSF	4–11-2016 serum	19–01-2017 serum
Enzygnost IgM index	0.07 (negative)			0.64 (negative)	0.14 (negative)
Enzygnost IgG index	0.12 (negative)			0.08 (negative)	0.12 (negative)
C6 peptide index	0.393 (negative)			0.15 (negative)	0.24 (negative)
Liaison IgM index	< 0.10 (negative)			< 0.10 (negative)	< 0.10 (negative)
Liaison IgG	< 5.0 AU/ml (negative)			12.4 AU/ml (equivocal)	14.7 AU/ml (equivocal)
IgM blot (Mikrogen)	Negative			Negative	Negative
IgG blot (Mikrogen)	Negative (only p41 +)			Negative (only p41 +)	Negative (only p41 +)
*Borrelia* PCR		Negative	Positive, *B. burgdorferi* s.l.)		
*B. miyamotoi PCR*		Negative	Negative		
*Pan-relapsing fever Borrelia PCR*		Negative	Negative		
Total IgA (g/l)	0.44 (reduced)			0.29 (reduced)	
Total IgM (g/l)	< 0.18 (reduced)			< 0.18 (reduced)	
Total IgG (g/l)	2.92 (reduced)			2.50 (reduced)	

A skin biopsy excluded sarcoidosis. Furthermore, the CSF was negative for all performed microbiological assays (Additional file 1: Table S2) and PCRs were negative for *Borrelia burgdorferi* sensu lato (s.l.), the tick-borne relapsing fever genus, as well as a species-specific PCR for *Borrelia miyamotoi* (Table 1, Additional file 1: Supplemental methods). Serology was negative for HIV and *Treponema pallidum* (Additional file 1: Table S2), and *Borrelia* IgM and IgG in serum were negative (Liaison, Diasorin S.p.A., Saluggia, Italy) (Table 1). The patient mentioned regular walks with his dog in the woods, but did not remember any tick bites.

During his admission, spontaneous resolution of symptoms made the patient refuse a brain biopsy and he was discharged on September 22nd without definite diagnosis. Due to a relapse of dysarthria, dysphagia, cognitive decline, ataxia and a novel paresis of the right facial nerve he was re-admitted October 5th. While a brain biopsy was already planned, a lumbar puncture was repeated and a PCR on *B. burgdorferi* s.l. turned out weakly positive (Ct value 37), confirmed by sequencing and PCR in a second laboratory. The patient was diagnosed with neuroborreliosis and was treated with ceftriaxone 2 g/d intravenously for one month. After an initial worsening of symptoms within the first two days, he experienced a recovery and was able to continue the final two weeks of treatment in a home care setting. Finally, minimal dysarthria and dysphagia were the only remaining symptoms after treatment.

After establishing the diagnosis of neuroborreliosis in this patient, we were interested whether other serologic tests would have been able to establish the diagnosis, or that this patient was truly seronegative (which is extremely rare). We also compared serology during (4–11-2016) and after treatment (19–01-2017). Serology was negative in the Enzygnost IgM and IgG ELISA (Siemens Healthcare Diagnostics GmbH, Marburg, Germany), C6 peptide ELISA (Immunetics Inc., Boston, MA, USA), and Liaison IgM in all sera, while the Liaison IgG became equivocal in intra- and post-treatment sera. Interestingly, both IgM and IgG blots (Mikrogen GmbH, Neuried, Germany) were negative, with only an IgG p41 band positive in all tested serum samples. A retrospective PCR on the skin biopsy failed to detect *Borrelia* DNA.

## Discussion and conclusions

It is generally accepted that patients with disseminated Lyme borreliosis with symptoms for more than six weeks reveal positive *Borrelia* serology [1, 2]. To our knowledge, this is the first neuroborreliosis case described in which a wide array of serologic assays for *Borrelia* remained negative, despite absence of treatment for over 16 weeks. Few other studies have suggested seronegative neuroborreliosis patients, however, lacking serologic analysis with more than one test [3], or described negative ELISA and blot after only 2 weeks of symptoms [4]. In our case, only P41 (flagellin) IgG was detected by blot and the Liaison IgG became equivocal 2 months after initial investigation, both of which would not have led to a clinical diagnosis of neuroborreliosis in this patient by any currently available diagnostic algorithm. P41 antibodies are often present in patients with neuroborreliosis [2, 5], although their specificity is subject of debate and P41 antibodies contribute the least to IgM or IgG blot scores [6]. In this case, antibody responses have probably been subdued due to use of rituximab, with a clinical picture similar to what has been described in other patients with neuroborreliosis caused by *B. burgdorferi* s.l. [3, 4] and *B. miyamotoi* [7, 8] after use of rituximab. Indeed, there was a lymphopenia in blood and total IgA/IgM/IgG concentrations were reduced during the entire disease episode, consistent with rituximab-mediated repression of B-cell mediated antibody production. In a humanized mouse model, it was previously shown that administration of rituximab impaired antibody responses against *Borrelia hermsii*, leading to a persistent spirochetemia [9].

Current diagnostic criteria of Lyme neuroborreliosis in regular patient categories require (1) neurological symptoms suggestive of LNB without other obvious explanations; (2) cerebrospinal fluid pleocytosis; (3) intrathecal antibody production against *Borrelia burgdorferi* s.l. [10].

We believe that in the patient population with an unexplained meningo-encephalitis and recent use of rituximab in Lyme borreliosis endemic areas, even without a history of tick bites, a PCR on CSF for both Lyme and relapsing fever *Borrelia* can be indicated in addition to testing for intrathecal antibody production. However, as this case illustrates, *Borrelia* PCR on CSF is notoriously insensitive (10–30%) [10, 11], and cannot fully exclude neuroborreliosis. In retrospect, a brain biopsy might have delivered an earlier diagnosis, but the patient refused. There are scarce reports on seronegative patients who do indeed have intrathecal antibody production, so we cannot fully exclude the presence of *Borrelia* antibodies in this patient [12, 13]. However, given the near-absence of B-cells in CSF, the lack of serum antibodies and the progressive meningo-encephalitic disease progress over months, we hypothesize that intrathecal antibodies were absent. Unfortunately though, a retrospective analysis of intrathecal antibodies or CXCL13 was impossible because no CSF was available due to extensive testing.

In hindsight, the skin lesions that appeared early in the disease process suggest he might have experienced atypical multiple erythema migrans lesions. A *Borrelia* PCR on his erythematous skin lesion however was negative, suggesting it was indeed a drug eruption or a para-infectious skin reaction. A trial of ceftriaxone was not performed in this patient, and it might be considered in patients when the clinical suspicion of neuroborreliosis is high enough and further diagnostic options are lacking. Nevertheless, this is the first case of neuroborreliosis to be negative in a variety of serologic assays after a protracted period of disease, suggesting this phenomenon might be extremely rare. Importantly, the uniqueness of this case warrants caution in extrapolating findings to patients with less severe humoral immune deficits. To conclude, this case illustrates the potential for persistently seronegative Lyme neuroborreliosis in patients with immunomodulatory therapy, and the need for further investigations or trial therapy in the rare case of a strong and persistent clinical suspicion for seronegative neuroborreliosis.

## Additional file

Additional file 1: **Table S1.** Cerebrospinal fluid cytology and chemistry. Table S2 Microbiologic diagnostic assays performed. Supplemental methods. (DOCX 26 kb)

## Abbreviations

B. b. s.l.: *Borrelia burgdorferi* sensu lato complex
CSF: Cerebrospinal fluid
LNB: Lyme neuroborreliosis
MRI: Magnetic resonance imaging
PCR: Polymerase chain reaction
PET-CT: Positron emission tomography – computed tomography
R-CHOP: Rituximab, doxorubicine, vincristine, cyclofosfamide, prednisone
