# Supplementary material for: Case report: persistently seronegative neuroborreliosis in an immunocompromised patient
Source: BMC Infect Dis. 2018 Aug 2;18:362. doi: 10.1186/s12879-018-3273-8 (PMC6090844; doi:10.1186/s12879-018-3273-8)
Supplement: Supplementary file 1 — Table S1. Cerebrospinal fluid cytology and chemistry. Table S2 Microbiologic diagnostic assays performed. Supplemental methods. (DOCX 26 kb) [file 12879_2018_3273_MOESM1_ESM.docx]

Table S1. Cerebrospinal fluid cytology and chemistry

|  | **25-8-2016** | **31-8-2016** | **7-10-2016** |
| --- | --- | --- | --- |
| **Leucocytes/μL** | 279 ↑ | 367 ↑ | 146 ↑ |
| **% B-cells** | 0.001 ↓ | 0 ↓ | 0 ↓ |
| **Erythrocytes/μL** | 7 ↑ | 5 ↑ | 0 |
| **Glucose mmol/L** | 1.5 | 1.4 | 1.4 |
| **Protein g/L** | 2.55 ↑ | 2.92 ↑ | 2.18 ↑ |

Table S2. Microbiologic diagnostic assays performed

| **Material** | **Date** | **PCR** | **Serology** | **Other** |
| --- | --- | --- | --- | --- |
| **Serum** | 25-08-2016 |  | HBsAg, a-HBc, HCV, HIV |  |
|  | 26-08-2016 |  | Treponema pallidum antibodies |  |
|  | 01-09-2016 |  | TBEV, LCMV |  |
| **CSF** | 25-08-2016 | HSV, VZV, enterovirus, CMV, EBV, JCV, Mycobacteria | Cryptococcal antigen | Gram, auramine |
|  | 31-08-2016 | *Trophyrema whipplei* |  | Gram: moderate leukocyte count, otherwise negative. |
|  | 07-10-2016 | HSV, EBV, mycobacteria |  | Gram: sporadic leukocytes, otherwise negative. Auramine. |

Supplemental methods

- *Borrelia* burgdorferi sensu lato PCR:

1. VU Medical Center: based on the OspA gene. 1500nM primers and 225nM probe as described in Gooskens et al. CMI 2006, performed in a lightcycler 480 II (Roche), using the following protocol: 2 minutes 50°C, 10 minutes 95°C and 45 cycles of [15 seconds 95°C and 1 minute 55°C].

2. Academic Medical Center: Based on the OspA gene. Previously described in Coumou et al CMI 2015.

- *Borrelia* genus PCR: based on the 16S rDNA gene, using the same methods as the *B. burgdorferi* s.l. PCR (as performed at the Academic Medical Center), using the following primers: Forward GGTAATACGTAAGGGGCGAG, Reverse CTAACTTCCYCTATCAGACTCTAG, Probe FAM- ATTGGGCGTAAAGGGTGAGTAGGCG –NFQ. Cycling conditions: 2 minutes 50°C, 10 minutes 95°C and 50 cycles of [15 seconds 95°C and 1 minute 60°C]. Sequence of the positive PCR product: GCGTAAAGGGTGAGTAGGCGGATATATAAGTCTATGCATAAAATACCACAGCTCAACTGTGGAACTATGTTGGAAACTATATGTCTAGAGTCTGATAGAGGAAGTTAGAC

- Relapsing fever *Borrelia* PCR: Previously described in Hovius et al. Lancet 2013
